# Supplementary material for: JMJD6 Autoantibodies as a Potential Biomarker for Inflammation-Related Diseases
Source: Int J Mol Sci. 2024 Apr 30;25(9):4935. doi: 10.3390/ijms25094935 (PMC11084951; doi:10.3390/ijms25094935)
Supplement: Supplementary file 1 [file ijms-25-04935-s001.zip › ijms-2942366-SI.pdf]

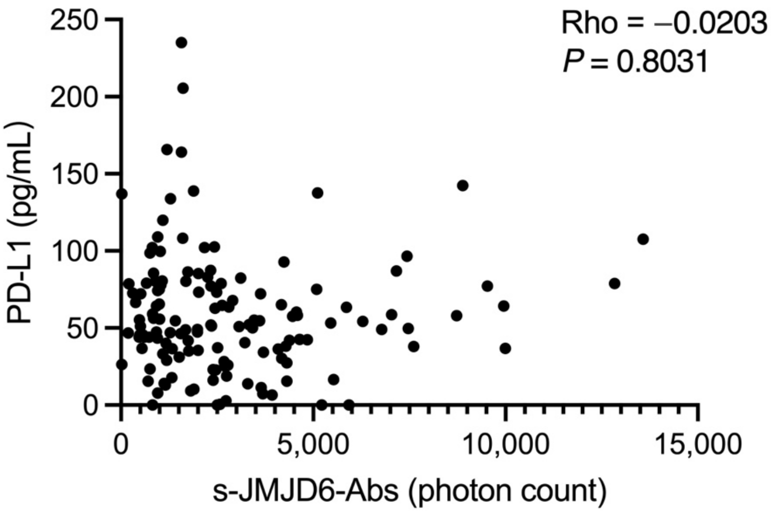

Supplementary Figure S1. Spearman correlation analysis between the levels of serum anti-JMJD6 antibodies (s-JMJD6-Abs) and PD-L1 in patients with esophageal cancer.
